# Outcomes of transconjunctival sutureless 27-gauge micro-incision vitrectomy surgery in diabetic vitreous haemorrhage

**DOI:** 10.12669/pjms.331.11617

**Published:** 2017

**Authors:** Bashiran Shahzadi, Syed Fawad Rizwi, Faisal Murtaza Qureshi, Kanwal Latif, Syed Asaad Mahmood

**Affiliations:** 1 Dr. Bashiran Shahzadi, FCPS. Department of Ophthalmology, LRBT Free Eye Hospital, Karachi, Pakistan; 2 Prof. Syed Fawad Rizwi, FCPS. Department of Ophthalmology, LRBT Free Eye Hospital, Karachi, Pakistan; 3 Dr. Faisal Murtaza Qureshi, FRCS. Department of Ophthalmology, LRBT Free Eye Hospital, Karachi, Pakistan; 4 Dr. Kanwal Latif, FCPS. Department of Ophthalmology, LRBT Free Eye Hospital, Karachi, Pakistan; 5 Dr. Syed Asaad Mahmood, FCPS. Department of Ophthalmology, LRBT Free Eye Hospital, Karachi, Pakistan

**Keywords:** 27-gauge micro-incision vitrectomy surgery, Diabetic retinopathy, Proliferative diabetic retinopathy, Vitreous hemorrhage

## Abstract

**Objective:**

To evaluate the visual outcomes of 27-gauge transconjunctival sutureless vitrectomy surgery and its complications in patients with diabetic vitreous hemorrhage.

**Methods:**

A quasi-experimental study was conducted where eighty seven eyes of 87 uncontrolled type II diabetes mellitus patients presenting with diabetic vitreous hemorrhage were selected to undergo 27-gauge transconjunctival sutureless micro-incision vitrectomy surgery. Main outcome measured was best corrected visual acuity (BCVA). Post-operative complications were also screened for at each visit. The follow ups were at post-operative day one, one month, three months and six months respectively.

**Results:**

Out of 87 patients, 52 (59.8%) were males and 35 (40.2%) were females. The mean age of the patients was 52.32 ± 6.78 years (95% CI: 53.13 - 55.57). For most of the patients, the BCVA improved progressively with each subsequent follow up visit. Pre-operative BCVA was 1.01 ± 0.206 logMar, compared to BCVA at final follow up of 0.44 ± 0.231 (p-value < 0.001). Six (6.9%) patients developed recurrent vitreous hemorrhage during the study period, four (4.6%) developed cataract, one (1.1%) had increased intraocular pressure and sub conjunctival hemorrhage was present in two (2.3%).

**Conclusion:**

27-gauge micro-incision vitrectomy surgery is an effective sutureless surgery with favorable outcomes, in terms of vision, in patients with diabetic vitreous hemorrhage. The associated complications are few which can be easily managed.

## INTRODUCTION

Diabetes mellitus is one of the most common medical problem in today’s world that causes a wide range of systemic complications. This has a substantial effect on the patient’s functioning in the society as the disease occurs in an individual’s most productive years.^1^ Patients with diabetes often develops ophthalmic complications such as cataract, glaucoma and distal neuropathies. However, diabetic retinopathy is the most common as well as the most blinding complication.^2^

In Unites States, diabetic retinopathy is the prime reason of blindness in people aged over 25 years of age. Proliferative diabetic retinopathy (PDR) is among the highest vision threatening complication, together with diabetic macular edema (DME).^3^ In Pakistan, the prevalence of PDR is reported around 2.65-5% in various studies.^4^,^5^ Among the major causes of visual impairment in patients of PDR, vitreous hemorrhage (VH) is the foremost cause of sudden visual loss.

Pan retinal photocoagulation was presumed to be the primary mode of treatment for PDR but recent advances have led to the use of instruments and surgical techniques for complications like vitreous hemorrhage and tractional retinal detachment (TRD) to be managed by vitrectomy.^6^

Retinal surgeries were revolutionizes by the invention of pars plana approach. The machine was first invented by Robert Machmer in which 1.5mm of sclerotomy was required.^7^ O’Malley and Heintz invented 3 ports sclerotomy for infusion, illumination and cutting and by separating these, the size of the sclerotomy was reduced.^8^ Further modifications established 20 gauge pars plana vitrectomy as the standard procedure for vitrectomy surgery.

Micro incision vitrectomy surgery (MIVS) has eased the vitrectomy surgery and offers numerous advantages over traditional 20-gauge surgery, such as reduced operating time, improved patient comfort, lower astigmatism and minimal conjunctival scarring.^9^

Glido Fujii et al. in 2002 introduced the current model of transconjunctival suture less MIVS with a trocar cannula system and 25 gauge instruments. Although with time, lack of instrument flexibility, poor illumination and low flow rates became the main concerns of 25 gauge MIVS.^10^ Eckhart with DORC presented a new suture less vitrectomy using 23 gauge instrumentation in 2003.^11^

In 2010, Oshima et al. introduced 27 gauge MIVS with 0.4mm incision size.^12^ The underlying motivation for this 27 gauge vitrectomy was that smaller is better. The 27 gauge incisions are 20% smaller than 25 gauge incisions and the wound construction is simpler. It is constructed with a one-step incision verses the techniques of angled incision used with 23 and 25 gauge instruments. Wound self-sealing is one of the advantages of 27 gauge surgery, it reduces risk of iris prolapse and permits faster wound healing.^12^

Moreover, the smaller wound size of 27 gauge MIVS causes less inflammation and less post-operative pain. Although indications for 27 gauge vitrectomy are increasing, a definite criteria for proper case selection is not present and the VR surgeon develops one based on his or her own preferences.

This study was designed to determine the visual outcomes after 27 gauge transconjunctival sutureless surgery for diabetic vitreous hemorrhage and assess any associated complications.

## METHODS

The study was conducted from January 2015 to July 2016 at LRBT tertiary care hospital. This was a prospective non comparative interventional study that was approved by hospital ethics committee. Patients were recruited using a non-probability convenience sampling technique. Known cases of diabetic retinopathy with non-resolving vitreous hemorrhage were included. Exclusion criteria were applied to screen out patients with history of any past ocular surgery, uveitis, glaucoma, cataract or retinal detachment.

The surgical procedure was briefed to all patients and written informed consent obtained after explaining the risks and benefits. Data collected for the study included the age of the study participants, gender, dates of admission, operation and discharge, investigations, and a note of any post-operative complications including cataract, hypotony, endopthalimitis and sub conjunctival hemorrhage.

The preoperative assessment consisted of testing visual acuity using the log MAR chart, anterior segment examination, cataract assessment, intraocular pressure (IOP) measurement with Goldmann applanation tonometer (Haag Streit AT 900), and dilated fundus exam using a +90 diopter lens. Additionally, blood pressure, HbA1c and fasting sugar levels were considered in systemic assessment.

Equipment used for surgery was Constellation Vision System (Alcon Laboratories Inc.), with 27-gauge infusion cannula, cutter, endoilluminator, endolaser probe and trocars. All the surgeries were done by the same surgeon with an experience in vitreoretinal surgery of over 10 years.

All cases were done under local anesthesia in which retrobulbar injection containing lidocaine and bupivacaine was given. After strict aseptic measures scleral tunnels were formed by trocar cannula which was inserted obliquely at an angle of 30° at superonasal, infratemporal and superotemporal area transconjunctivally, at a distance of 4.00mm from limbus. The infratemporal cannula was used for the infusion line while the illumination and vitrectomy cutter were introduced throught the superior cannulas. Complete vitrectomy was done, followed by pan retinal photocoagulation (PRP) by endolaser. Superior cannulae were removed at the end of surgery, taking care to confirm conjunctival repositioning over the sclerotomies. Infratemporal cannula connected with infusion line was detached last and conjunctival repositioning confirmed. The follow ups visits were at postoperative day one, after one week, one month, three months and last follow-up at six months. BCVA was assessed by a trained optometrist with a logMAR chart.

Complications were screened for during the postoperative visits. These were recorded and managed accordingly. IBM SPSS Statistics 21 was used to analyze the data on BCVA by means of paired t- test using 95% confidence interval. A p-value of ≤ 0.01 was considered statistically significant.

## RESULTS

Eighty seven eyes (87 patients) underwent 27-gauge MIVS and their data analyzed. Mean age range was 52.32±6.7 years (range 40-60 years), of which 52 (59.8%) were males and 35 (40.2%) were females.

Pre-operative BCVA was 1.01 ± 0.206 logMAR, which subsequently progressed to 0.44 ± 0.231 at the last follow-up (p-value < 0.001). Comparison was done between pre-operative BCVA and the BCVA on post-operative day one, one month’s and six months’ follow-up. Paired sample t-test was run on all 3 pairs with 95% confidence interval and showed a p-value < 0.001 in each case. The statistical analysis of BCVA preoperatively and at 6 months post-operative visit is presented in Table-I.

**Table-I:** Paired Sample Test of Difference in Preoperative and Final BCVA.

Difference in BCVA*	Mean	Std. Deviation	95% Confidence Interval (Lower)	95% Confidence Interval (Upper)	p-value
	0.5747	0.1984	0.0213	0.5324	< 0.000

* Difference in BCVA = Preoperative BCVA (logMAR) – BCVA at 6 months (logMAR)

The post-operative complications which were managed on individual basis are shown in Table-II. Those with recurrent vitreous hemorrhage underwent a redo surgery via 27 gauge after a gap of several months.

**Table-II:** Complications frequency table.

Complication	Frequency(n)	Percent (%)
Cataract	4	4.6
Increased IOP	1	1.1
Sub-conjunctival hemorrhage	2	2.3
Vitreous Hg	6	6.9

Total	13	

## DISCUSSION

Par plana vitrectomy is considered to be the procedure of choice in cases of non-clearing vitreous hemorrhage.^13^ Cutting efficiency of small-gauge vitrectomy probes has been enhanced with the newer vitrectomy machines.^14^

Surgical wounds are based on a theory that “much smaller is much better.” In fact, Japanese surgeons radically shifted from 20 gauge to 25 gauge systems over the years along with the development of better instrumentation and more efficient vitrectomy machines.^15^ In 2008, 27-guage system was used for macular disease and simple vitreous hemorrhage and showed both the anatomical and visual results were encouraging.^16^

Our study shows considerable improvement in BCVA after six months post-MIVS. Major improvement was observed in BCVA on the 1^st^ post op day and at the end of the first post-operative month, while small improvements continued to be seen up to six months post-operatively. Oshima Y did a study which showed mean BCVA changes 0.46±0.51.^17^ The 27-gauge system not only simplifies procedures and shortens the operating time but it also eliminates the wound sealing problems which are sometimes seen with 23 and 25 gauge machines. Full spectrum of vitreoretinal diseases can easily be treated with newer 27-gauge instruments.^17^

The chandelier lighting system is a new tool for bimanual vitrectomy, which uses 25 gauge and 27 gauge format or a 29 gauge endoillumination.^18^ Suture less vitrectomy has been reported to increase the incidence of postoperative hypotony in literature.^19^ Hypotony (IOP ≤ 5mmHg) did not occur in any patient in our study. A study by Oshima Y et al. study showed all sclerotomies to have self-sealed without hypotony.^16^

Khan MA et al. conducted a study which showed 5 eyes (5.3%) with transient hypotony, 8 eyes (8.4%) with transient ocular hypertension, and 5 eyes (5.3%) with vitreous hemorrhage as post-operative complications. There was no postoperative endophthalmitis, retinal tear secondary to sclerotomy, or choroidal detachment.^20^ Recurrent vitreous hemorrhage occurred in five cases in our study due to underlying basic pathology of PDR, and these were managed with a re-do surgery. Intra ocular hypertension (IOP >22 mmHg) developed in two patient and was treated successfully with oral anti glaucoma medicine.

## CONCLUSION

27-gauge micro-incision vitrectomy surgery is an effective sutureless surgery with favorable outcomes, in terms of vision, in patients with diabetic vitreous hemorrhage. The associated complications are few which can be easily managed.
